# From memorisation to reasoning: a qualitative study of first-year medical students’ cognitive shifts in computer science education

**DOI:** 10.3389/fmed.2026.1822748

**Published:** 2026-04-29

**Authors:** Omar Alobud

**Affiliations:** 1 King Abdullah International Medical Research Center, Riyadh, Saudi Arabia; 2 King Saud bin Abdulaziz University for Health Sciences, Riyadh, Saudi Arabia

**Keywords:** cognitive strategies, medical education, memorisation, problem-solving, qualitative research

## Abstract

**Background:**

Many students enter medical school having achieved prior academic success primarily through memorisation-based learning strategies. This study explored how first-year medical student’s shift from memorisation-based approaches toward problem-solving and reasoning-based strategies when encountering an introductory computer science course that resists recall-oriented learning.

**Methods:**

A qualitative interview study was conducted with 12 first-year medical students enrolled in a mandatory computer science course. Semi-structured interviews explored students’ learning approaches, experiences when familiar strategies proved inadequate, and development of alternative cognitive strategies. Data were analysed using reflexive thematic analysis.

**Results:**

Four interconnected themes emerged: reliance on memorisation at course entry, early failure using recall-based strategies, shift toward stepwise problem-solving, and increased focus on process over correct answers. Students initially attempted to memorise programming syntax and examples as they would any other academic content, but discovered that recall did not translate into the ability to write functioning code. Through repeated failures and iterative experimentation, students gradually developed approaches emphasising logical reasoning, systematic problem decomposition, and constructive engagement with tasks rather than reproduction of memorised information.

**Conclusion:**

The shift from memorisation to problem-solving represents a fundamental reconceptualisation of what learning involves, rather than simply acquiring new study techniques. Programming tasks inherently resist memorisation strategies, creating conditions where cognitive adaptation becomes necessary. Medical curricula should consider incorporating early learning experiences that require reasoning and problem-solving rather than recall, helping students develop cognitive flexibility before encountering high-stakes clinical contexts.

## Introduction

Many students enter medical school having achieved prior academic success primarily through learning strategies that emphasise memorisation, careful note-taking, and accurate reproduction of information. These approaches prove highly effective in lecture-based environments assessed through recall examinations. However, such strategies often develop implicitly, becoming habitual patterns applied without conscious awareness of their limitations (1, 2). Students who consistently succeed through memorisation rarely need to question whether alternative approaches might be necessary. Consequently, many high-achieving students arrive at medical school with a relatively narrow learning repertoire, optimised for content absorption but less developed for tasks requiring reasoning, problem-solving, or navigating ambiguity (3). This narrowness becomes problematic when students encounter contexts where success depends not on remembering information but on applying logic, constructing solutions, and working through problems without predetermined answers (4).

Problem-solving represents a qualitatively different cognitive demand from memorisation. While memorisation involves encoding and retrieving information, problem-solving requires analysing situations, identifying relevant principles, generating approaches, testing them, and adjusting based on feedback (5, 6). In problem-solving contexts, errors become sources of valuable feedback rather than failures to avoid. Furthermore, problem-solving often involves ambiguity and uncertainty, where multiple approaches may be viable and evaluating adequacy requires judgment rather than comparison against a memorised standard (7). Students accustomed to authoritative textbook answers often find problem-solving deeply disorienting precisely because the authority shifts to their own reasoning processes.

This study is theoretically grounded in constructivist and experiential learning frameworks. Constructivist theory holds that learners actively build understanding through engagement with challenging tasks, rather than passively receiving information (7). Kolb’s experiential learning cycle similarly emphasises that durable learning emerges through cycles of concrete experience, reflection, and active experimentation (5). Both frameworks suggest that when memorisation strategies fail to transfer, the resulting breakdown creates conditions where cognitive adaptation becomes necessary rather than optional (8, 9). This adaptation typically occurs not through systematic planning but through iterative experimentation, as students try different approaches, observe results, and gradually build competence through accumulated experience (10).

Computer science education provides an ideal context for observing this shift because programming inherently resists memorisation while demanding logical reasoning and iterative refinement. Programming requires students to decompose problems into logical steps, debug code by analysing errors, and iterate toward solutions through multiple attempts (11). These demands differ fundamentally from memorising biological pathways or anatomical terminology, making the inadequacy of recall-based strategies immediately apparent (12). The learning environment itself makes this mismatch visible quickly, as non-functioning code provides immediate feedback that familiar strategies are insufficient.

The present study therefore aims to explore how first-year medical students describe changes in their learning approaches when engaging with problem-solving tasks in an introductory computer science course, examining what specific shifts occur and what prompts these changes.

## Methods

### Design

This study employed a qualitative research design to explore how first-year medical students describe and experience changes in their learning approaches when engaging with problem-solving tasks in an introductory computer science course. Qualitative methodology was chosen because the research questions focus on subjective perception and interpretation—specifically, how students conceptualise their own learning processes and make sense of changes in cognitive strategies—which cannot be adequately captured through quantitative measures alone.

### Participants and sampling

Twelve first-year medical students enrolled in a mandatory introductory computer science course participated in this study. All were in their first semester and had no formal background in computer science or programming, ensuring they were genuinely encountering problem-solving demands that differed substantially from prior educational experiences. Participants were recruited through course announcements, with purposive sampling used to ensure diversity in academic background and self-reported confidence about learning new subjects.

In qualitative research, sample size adequacy is determined not by statistical power but by the richness of data and the achievement of informational sufficiency (13). Twelve participants are consistent with established norms for reflexive thematic analysis with a clearly bounded participant group and focused research questions. Data collection and analysis proceeded concurrently, with the interview guide refined iteratively and data sufficiency assessed throughout the process.

### Data collection

Individual semi-structured interviews were conducted after students had completed approximately three-quarters of the computer science course. This timing allowed participants substantial exposure to programming tasks while ensuring experiences remained sufficiently recent to be described in detail. Interviews lasted between 50 and 75 min and were conducted in quiet, private locations. The interview guide began with open questions inviting participants to describe their usual study methods and how those methods worked when applied to programming. Follow-up questions explored specific instances when usual strategies proved inadequate, what participants tried differently, and whether they perceived broader changes in how they thought about learning. All interviews were audio-recorded with explicit participant consent and transcribed verbatim, preserving exact language as these details often revealed important nuances in how students conceptualised their learning.

### Analysis

Interview transcripts were analysed using reflexive thematic analysis (13), an approach that recognises theme development as an active interpretive process rather than passive discovery of pre-existing patterns. Coding was conducted inductively, with codes generated directly from participant accounts rather than applied from a predetermined framework. Analysis proceeded through iterative phases: immersive reading of all transcripts, systematic generation of initial codes across the dataset, collation of coded segments into candidate themes, and recursive review and refinement of themes until a coherent analytic account was produced.

To enhance rigour, the author maintained a reflexivity journal throughout data collection and analysis, documenting assumptions and analytic decisions. Ethical approval was obtained from the Institutional Review Board. All participants provided written informed consent prior to interview.

## Results

Analysis revealed four interconnected themes capturing how first-year medical students shifted from memorisation-based approaches toward problem-solving and reasoning-based strategies. These themes illustrate a progression from initial reliance on recall-oriented methods, through recognition of their inadequacy, to gradual development of alternative approaches emphasising logical thinking, iterative experimentation, and process-oriented engagement.

### Theme 1: reliance on memorisation at course entry

Nearly all students described entering the computer science course with confidence that they could master programming by memorising syntax, studying examples, and reproducing demonstrated procedures—the same strategies that had secured their admission to medical school. One student spent hours creating detailed notes from lectures, highlighting syntax rules, and reviewing them repeatedly before attempting assignments, fully expecting that thorough memorisation would enable them to write code successfully. Another made flashcards of programming commands and their definitions, feeling prepared based on their ability to recall this information accurately.

Students articulated an implicit theory of learning in which understanding meant knowing information. As one participant explained:

“I studied it the same way I always study. I read it, I highlighted it, I made sure I could remember it. That’s what learning is” (Participant 3).

This assumption was so fundamental that most participants did not recognise it as an assumption at all, but simply as how learning worked. The computer science course represented just another subject to be absorbed and reproduced.

### Theme 2: early failure using recall-based strategies

Students described a jarring experience when they discovered that memorising programming concepts and syntax did not translate into the ability to write functioning code. Participants recounted sitting down to complete assignments after thorough preparation, only to find themselves unable to begin because problems presented did not match memorised examples closely enough to reproduce a known solution.

One participant described a particularly telling moment of failure:

“I knew the syntax, but I didn’t know what to do with it. I could tell you exactly what a loop is. I could not write a loop that actually solved anything” (Participant 4).

Another participant explained that they could recite definitions of loops and conditionals perfectly but found themselves paralysed when deciding whether a particular problem required a loop, what kind, and how to structure it. Knowledge of individual components did not automatically combine into the ability to use those components to build solutions.

Students described this as unlike anything encountered in other subjects, where additional studying typically improved performance. In programming, more memorisation did not help. This failure forced students to confront the possibility that their fundamental approach to learning might be inadequate—a realization that was both cognitively confusing and emotionally challenging, as it directly threatened their identity as competent learners.

### Theme 3: shift toward stepwise problem-solving

Students gradually began developing approaches that emphasised breaking problems into manageable steps, reasoning through logic systematically, and constructing solutions incrementally. This shift did not occur through explicit instruction but emerged from necessity as memorisation proved insufficient (14). Several participants described beginning to write out problems in plain language before attempting to code—forcing themselves to articulate exactly what the program needed to do step by step (15).

One student described a concrete shift in practice:

“I started drawing boxes and arrows before I wrote any code. It felt slow but it actually worked—way better than trying to remember examples” (Participant 7).

Another participant described learning to tackle small pieces of problems independently—writing code to handle one specific aspect, testing whether that piece worked, then adding the next piece—rather than attempting to construct entire solutions at once. Students articulated a fundamental shift in cognitive orientation: instead of asking ‘what example does this match?’ they began asking ‘what does this problem require logically?’ and ‘what steps would solve this in sequence?’

### Theme 4: increased focus on process over correct answers

Students described a changing understanding of what it meant to learn effectively. One participant explained:

“At first I felt like every error meant I’d failed. Then I realised the errors were the lesson. Getting through them was the actual learning” (Participant 9).

Another student described realising that when they successfully completed a programming assignment, what they had learned was not the specific solution to that problem but rather a way of thinking through similar problems—a process applicable to new situations rather than a fact to store and retrieve. Students also described increased attention to understanding why code worked rather than simply getting it to work, recognising that comprehending the underlying logic was more valuable than producing functioning programs through trial and error without understanding.

This shift reflected a deeper reconceptualisation of learning itself: from accumulating correct information to developing cognitive processes and reasoning capabilities applicable across varied problems.

## Discussion

The findings demonstrate a genuine shift in how students approached learning, moving from memorisation-based strategies toward reasoning-oriented approaches emphasising logical thinking, systematic problem decomposition, and iterative construction of solutions. This transition emerged because programming tasks resisted recall-based methods in fundamental ways, creating conditions where adaptation became necessary. Consistent with constructivist and experiential learning frameworks, students did not shift through a single insight or explicit instruction, but through persistent engagement with tasks that required iteration and reflection (5, 9, 16).

The cognitive difficulty students experienced when memorisation strategies proved inadequate played a crucial role in prompting this shift. Repeated failure disrupted automatic, habitual patterns and created pressure to experiment with alternatives (10). This is consistent with productive failure theory, which suggests that well-calibrated difficulty can promote deeper learning by exposing the limitations of existing strategies (17). The computer science tasks were challenging enough to resist memorisation, yet accessible enough that students could make progress through reasoning and iteration rather than becoming overwhelmed (7).

A particularly important aspect of how students developed problem-solving approaches was the iterative, practice-based nature of that development. Writing code, encountering errors, analysing what went wrong, testing hypotheses, and working toward functioning solutions created fundamentally experiential learning cycles (18). Each cycle contributed incrementally to students’ developing understanding, building tacit knowledge through accumulated practice rather than abstract comprehension of problem-solving principles. The social dimension also mattered: some students noted that working alongside peers and comparing debugging strategies helped normalise the experience of struggling through multiple attempts (19).

These findings carry important implications for medical curricula. If many students arrive with learning strategies optimised for memorisation, early curriculum experiences should deliberately incorporate tasks that require reasoning and problem-solving rather than recall, helping students develop cognitive flexibility before encountering high-stakes clinical situations (20). This does not mean abandoning systematic instruction, but thoughtfully designing tasks that resist pure memorisation and demand systematic thinking. Such experiences might include problem-based learning scenarios without predetermined solutions, clinical case analyses requiring integration of knowledge, simulation exercises, or collaborative projects requiring collective reasoning. The key is creating contexts where recall-oriented thinking is insufficient, where this is surfaced through appropriate feedback, and where students develop explicit awareness that different types of tasks demand different cognitive strategies (21–23).

## Conclusion

This study explored how first-year medical students shifted from memorisation-based learning toward problem-solving and reasoning-based strategies when engaging with an introductory computer science course. Programming tasks resisted familiar approaches in ways that made their inadequacy immediately apparent, prompting students to experiment with alternatives and gradually develop more reasoning-oriented strategies. This shift reflected a deeper reconceptualisation of learning itself—from absorbing information to developing cognitive processes applicable across varied problems.

These findings suggest that early medical curriculum experiences should deliberately include tasks requiring reasoning and problem-solving rather than recall, supported by appropriate scaffolding and feedback, to help students develop adaptive cognitive approaches before encountering high-stakes clinical contexts.

Future research should examine how shifts in learning approach developed in educational contexts like computer science transfer to clinical learning environments, and whether early development of problem-solving capabilities predicts adaptive capacity in later training stages. Investigating individual variation in how students navigate this cognitive transition, and exploring specific pedagogical approaches that best support reasoning-based learning strategies, would further strengthen understanding of how to cultivate cognitive flexibility throughout medical education.

## Data Availability

The raw data supporting the conclusions of this article will be made available by the author, without undue reservation.
